# Pharmacokinetics of S-1 monotherapy in plasma and in tears for gastric cancer patients

**DOI:** 10.1007/s10147-018-01387-6

**Published:** 2019-04-22

**Authors:** Hirofumi Yasui, Takeshi Kawakami, Hiroya Kashiwagi, Keita Mori, Katsuhiro Omae, Jun Kasai, Kunihiro Yoshisue, Masahiro Kawahira, Takahiro Tsushima, Nozomu Machida, Akira Fukutomi, Ken Yamaguchi

**Affiliations:** 1 Division of Gastrointestinal Oncology, Shizuoka Cancer Center, 1007 Shimonagakubo, Nagaizumi-cho, Sunto-gun, Shizuoka, 411-8777 Japan; 2 Division of Ophthalmology, Shizuoka Cancer Center, Shizuoka, Japan; 3 Clinical Research Center, Shizuoka Cancer Center, Shizuoka, Japan; 4 Medical Affairs Department, Taiho Pharmaceutical Co., Ltd., Tokyo, Japan; 5 Pharmacokinetics Research Laboratories, Taiho Pharmaceutical Co., Ltd., Tsukuba, Japan; 6 Shizuoka Cancer Center Hospital and Research Institute, Shizuoka, Japan

**Keywords:** S-1, Watering eyes, Gastric cancer, Pharmacokinetics

## Abstract

**Background:**

S-1 is an oral anticancer drug composed of tegafur (FT), which is a prodrug of 5-FU, 5-chloro-2,4-dihydroxypyridine (CDHP), and potassium oxonate. Recently, some studies have been reported on watering eyes caused by S-1. However, the mechanism of watering eyes caused by S-1 is still unclear. The aim of this study was to investigate the correlation between tears and plasma concentrations of FT, 5-FU, and CDHP, which are components and active modulator of S-1.

**Methods:**

We prospectively investigated the pharmacokinetics (PK) of FT, 5-FU, and CDHP in plasma and in tears of gastric cancer patients who were treated with S-1 monotherapy at the dose of 80 mg/m^2^/day. Plasma and tears from both eyes were obtained 1, 2, 4, and 8 h after S-1 administration on day 1 and 14 of the first cycle.

**Results:**

Total of eight patients were enrolled. All the FT, 5-FU and CDHP were detected both in plasma and in tears, and their PK parameters were measured. There was a positive correlation between the concentrations of FT, 5-FU and CDHP in the plasma and those in the tears on day 1 and day 14 (correlation coefficients *r*, right eye/left eye: *r* = 0.882/0.878, 0.877/0.890, and 0.885/0.878, respectively).

**Conclusion:**

There was a positive correlation between the concentrations of FT, 5-FU and CDHP in the plasma and those in the tears. The result is expected to facilitate the further investigation into the causes of watering eyes and the establishment of the effective methods for the prevention and the treatment.

## Introduction

S-1 is an oral anticancer drug which is composed of tegafur, gimeracil, and oteracil in a molar ratio of 1:0.4:1 [1]. S-1 plus cisplatin or oxaliplatin combination therapy has been recognized as a standard treatment for chemonaïve advanced gastric cancer (AGC) patients [2–4], and adjuvant S-1 monotherapy is a standard care for stage II and III gastric cancer after curative surgery with D2 dissection in Japan [5]. Thus, S-1 is a key drug widely administered for the treatment of both metastatic and resectable gastric cancer.

We previously demonstrated that Grade 2 or 3 watering eyes was observed in 25% of gastric cancer patients who received S-1 as adjuvant treatment for 1 year [6]. Recently, watering eyes is recognized as a common adverse event for S-1. Several reports have been published so far which report that disorder of lachrymal duct was observed among the patients orally taking S-1 and that 5-FU was detected in the tears of the patients with watering eyes, but these researches reported that FT, 5-FU, and CDHP, which are components and active modulator are transferred to tears, they did not measure the time-dependent changes of their concentrations in tears, and the mechanism of watering eyes caused by S-1 is still unclear [7–14].

In this study, we examined the correlation of the concentrations of FT, 5-FU, and CDHP in plasma and those in tears.

## Patients, materials, and methods

This study was open-label and single arm study which evaluated the PK of FT, 5-FU, and CDHP in plasma and tears, which was conducted in accordance with the Helsinki Declaration. The protocol was approved by the ethics committee of Shizuoka Cancer Center and written informed consents were obtained from all the patients before their participation in the study.

This study is registered to UMIN-CTR [http://www.umin.ac.jp/ctr/] (000021610).

### Eligibility

Criteria for the patient enrollment in the study included (1) histologically confirmed gastric or gastroesophageal junction adenocarcinoma, (2) no prior treatment with S-1, (3) creatinine clearance (Cockcroft-Gault formula) of 60 mL/min or higher, and (4) age 20–80 years.

Patients were excluded if they met any of the following exclusion criteria; (1) with synchronous and metachronous double cancer or multiple cancer, (2) with eye diseases or their previous history, (3) judged to be ineligible for pre-consultation by an ophthalmologist (4) uses contact lenses (5) has dry eye cases, and (6) with sinusitis or a nose trauma.

### Treatment

S-1 was administered orally twice a day for 28 days, followed by 14-day rest. The dose of S-1 administered per day was based on the patient’s body surface area (BSA) as follows: 80 mg for BSA less than 1.25 m^2^, 100 mg for BSA between 1.25 and 1.5 m^2^, 120 mg for BSA larger than 1.5 m^2^.

Dose reduction, delay of the treatment course, and change of the treatment schedule were decided at the discretion of the physicians. Generally, physicians considered S-1 dose reduction and/or schedule modification to 3-week treatment course (administration for 14 days followed by 7 days of rest) if patients experienced any grade 3 or higher hematological adverse events or unacceptable grade 2 or higher non-hematological adverse events.

### Pharmacokinetics

Peripheral blood samples (2 mL) were collected in heparinized tubes at 1, 2, 4, 8 h on day 1, and at 0, 1, 2, 4, 8 h on day 14 after S-1 administration during the cycle 1. Those samples were centrifuged at 3000 rpm for 15 min at 4 °C to prepare the plasma samples. Tears samples (10 mm to 30 mm) were collected by Color Bar Schirmer Tear Test strips (Eagle Vision, Inc., US) before S-1 administration and at 1, 2, 4, 8 h on day 1, and at 0, 1, 2, 4, 8 h on day 14 of the cycle 1. Schirmer strips samples and plasma samples were stored at − 60 °C until their analysis. The concentrations of FT, 5-FU, and CDHP in the plasma and tears were measured at FALCO Biosystems Ltd. (Kyoto, Japan) by a validated method using liquid chromatography with tandem mass spectrometry. For the measurement of FT, 5-FU and CDHP concentrations in tears, each sample was prepared by eluting the Schrimer strip with plasma. The volume of tears was calculated by converting millimeter on the Schrimer strip to microliter of tears using the standard curve.

### Statistical analysis

The following PK parameters were calculated for each patient using a non-compartmental model using the Phoenix®WinNonlin® ver. 6.4 (Certara, NJ, USA). The maximum concentration (*C*_max_) was the observed maximum concentration of FT, 5-FU, and CDHP in plasm and tears during the 8 h, and the time-to-the-maximum concentration (*T*_max_) was the time when the concentration reached *C*_max_. The area under the concentration–time curve from time zero to 8 h point (AUC_0−8_) was calculated using the linear trapezoidal rule and the half-life (*T*_1/2_) was determined by linear regression of the log-linear proportion of the concentration–time profile. Correlations between log-transformed concentrations of plasma and tears of each the FT, 5-FU, and CDHP on day 1 and day 14 were investigated by linear regression analyses (JMP ver. 9.0 software, SAS Institute Inc., Cary, NC, USA).

## Results

### Patients’ characteristics

Total of eight patients were enrolled. Patient characteristics are shown in Table 1. The median age was 68.5 years (range 40–76 years). The all patients were a good performance status (ECOG PS0) after gastrectomy. Four patients (50%) underwent distal gastrectomy, three underwent total gastrectomy and one underwent partial gastrectomy. The median creatinine clearance was 78.0 mL/min (range 63.0–119.7 mL/min).

**Table 1:** Baseline characteristics

	Number of Pts (%)
Median age, years (range)	68.5 (40–76)
Sex	
Male	7 (87.5)
Female	1 (12.5)
ECOG performance status	
0	8 (100)
1	0 (0)
Characteristics of disease	
Resected	8 (100)
Advance/metastatic	0 (0)
Gastric surgery	
Total gastrectomy	3 (37.5)
Distal gastrectomy	4 (50.0)
Partial gastrectomy	1 (12.5)
Median creatinine clearance, mL/min (range)	78.0 (63.0–119.7)

### Concentration–time profiles of FT, 5-FU, CDHP in plasma and tears at days 1 and 14

**Fig. 1:**
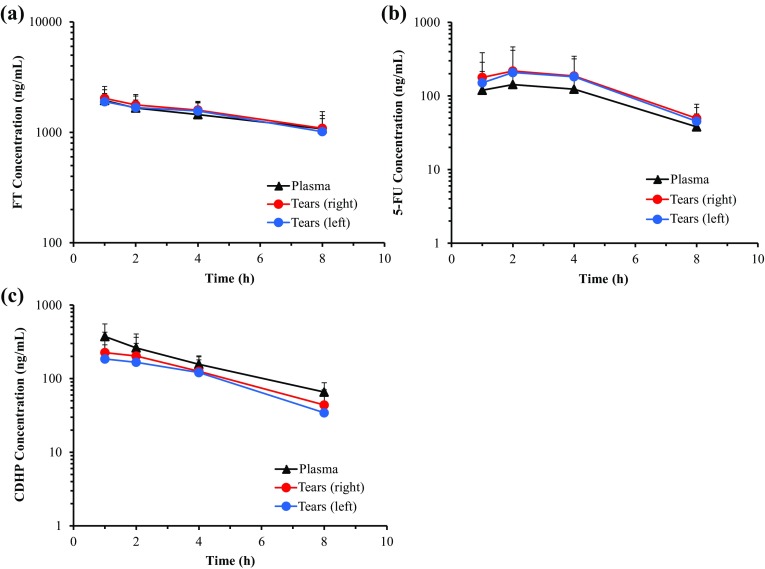
Concentration–time profiles of **a** FT, **b** 5-FU, **c** CDHP in plasma and tears at day 1. Mean(± SD) concentrations (ng/mL) at 1, 2, 4, 8 h after S-1 administration are shown. FT, tegafur; 5-FU, 5-fluorouracil; CDHP, 5-chloro-2,4-dihydroxypyridine

**Fig. 2:**
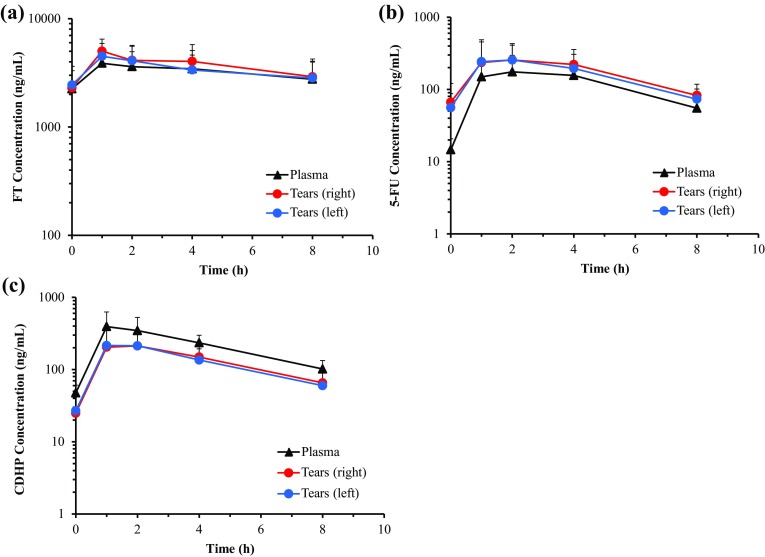
Concentration–time profiles of **a** FT, **b** 5-FU, **c** CDHP in plasma and tears at day 14. Mean (± SD) concentrations (ng/mL) at 0, 1, 2, 4, 8 h after S-1 administration are shown. FT, tegafur; 5-FU, 5-fluorouracil; CDHP, 5-chloro-2,4-dihydroxypyridine; SD, standard deviation

The concentrations of FT, 5-FU and CDHP in plasma and tears on days 1 and 14 are shown in Figs. 1 and 2, and PK parameters on days 1 and 14 are shown in Tables 2 and 3. All the FT, 5-FU and CDHP were detected both in plasma and in tears, and their PK parameters were measured. The PK parameters of FT, 5-FU and CDHP in plasma were consistent with the previous reports and the concentration–time profiles of FT, 5-FU and CDHP in tears were parallel to those in plasma. The concentrations of 5-FU in the tears tended to be higher than those in the plasma. On the other hand, the concentrations of CDHP in the tears to be lower than those in the plasma.

**Table 2:** PK parameters of FT, 5-FU and CDHP in plasma and tears on day 1

	*T*_max_ (h)	*T*_1/2_ (h)	*C*_max_ (ng/mL)	AUC_0−8_ (ng h/mL)
FT				
Plasma	1.4 ± 1.1	8.5 ± 3.5	2046.2 ± 269.3	10,786 ± 2556
Tears (right)	1.6 ± 1.1	9.9 ± 7.4	2155.5 ± 434.7	11,499 ± 1785
Tears (left)	1.9 ± 1.4	10.0 ± 9.2	2066.0 ± 334.2	11,000 ± 1808
5-FU				
Plasma	2.8 ± 1.0	2.6 ± 0.8	148.1 ± 91.8	768.5 ± 445.7
Tears (right)	2.9 ± 1.2	2.7 ± 1.3	232.9 ± 237.5	1145.7 ± 1063.3
Tears (left)	3.1 ± 1.2	2.6 ± 1.4	228.4 ± 200.7	1084.4 ± 855.2
CDHP				
Plasma	1.5 ± 1.1	3.0 ± 0.7	376.5 ± 173.4	1340.4 ± 423.1
Tears (right)	1.9 ± 1.4	2.8 ± 1.1	240.9 ± 191.6	979.4 ± 735.4
Tears (left)	1.9 ± 1.4	2.7 ± 1.1	213.9 ± 120.5	840.2 ± 492.3

*FT* tegafur; *5-FU* 5-fluorouracil; *CDHP* 5-chloro-2,4-dihydroxypyridine; *C*_*max*_ Maximum concentration; *T*_*max*_ Time-to-the-maximum concentration; *T*_*1*/*2*_ Elimination half-life; *AUC*_*0*−*8*_ Area under the concentration–time curve from time zero to 8 h point

**Table 3:** PK parameters of FT, 5-FU and CDHP in plasma and tears on day 14

	*T*_max_ (h)	*T*_1/2_ (h)	*C*_max_ (ng/mL)	AUC_0−8_ (ng h/mL)
FT				
Plasma	1.4 ± 1.1	11.4 ± 4.3	4221.5 ± 1584.8	26,108 ± 11,759
Tears (right)	1.7 ± 1.2	11.6 ± 11.2	5177.9 ± 1580.2	30,945 ± 12,064
Tears (left)	1.8 ± 1.2	11.4 ± 6.1	4826.7 ± 1327.2	27,787 ± 9924
5-FU				
Plasma	2.9 ± 1.1	3.1 ± 1.1	190.2 ± 103.0	1027.8 ± 523.4
Tears (right)	2.3 ± 1.4	5.4 ± 5.8	281.1 ± 194.9	1482.6 ± 989.1
Tears (left)	2.3 ± 1.4	3.8 ± 2.1	301.9 ± 220.0	1355.9 ± 816.2
CDHP				
Plasma	1.6 ± 1.1	3.6 ± 1.6	442.0 ± 171.4	1859.8 ± 594.5
Tears (right)	1.8 ± 1.2	2.6 ± 0.3	227.5 ± 116.6	1017.3 ± 499.0
Tears (left)	1.8 ± 1.2	2.4 ± 0.4	254.6 ± 143.8	979.0 ± 406.1

*FT* tegafur; *5-FU* 5-fluorouracil; *CDHP*; 5-chloro-2,4-dihydroxypyridine; *C*_*max*_ Maximum concentration; *T*_*max*_ Time-to-the-maximum concentration; *T*_*1*/*2*_ Elimination half-life; *AUC*_*0*−*8*_ Area under the concentration–time curve from time zero to 8 h point

### Correlations between tears and plasma concentrations of FT, 5-FU and CDHP

**Fig. 3:**
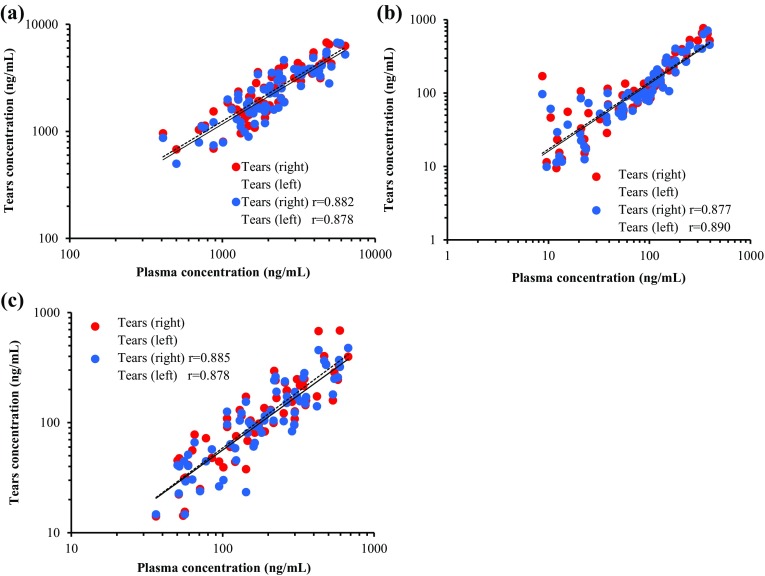
Correlation between tears and plasma concentrations of **a** FT, **b** 5-FU and **c** CDHP on day 1 and day 14. FT, tegafur; 5-FU, 5-fluorouracil; CDHP, 5-chloro-2,4-dihydroxypyridine; r, correlation coefficient

The correlation between the concentrations of FT, 5-FU and CDHP and those in tears on day 1 and day 14 are shown in Fig. 3. There was a positive correlation between the concentrations of all the FT, 5-FU and CDHP in the plasma and those in the tears (correlation coefficients *r*, right eye/left eye: *r* = 0.882/0.878, 0.877/0.890, and 0.885/0.878, respectively).

## Discussion

This study was the first report to clarify the time-dependent changes of drug concentrations in tears. There was a positive correlation between the concentrations of the FT, 5-FU and CDHP in tears and those in plasma.

Advantage of the administration of S-1 in adjuvant chemotherapy after curative surgery for Stage II and III gastric cancer was validated in ACTS-GC clinical study in 2007, and the number of patients who receive S-1 for as long as 1 year has been increased since then, in particular in Japan. Along with the increase, watering eyes and disorder of lachrymal duct have been observed more frequently in these days, and it has been drawn attention as an adverse event of S-1.

S-1 is one of the key drugs widely administrated not only for the adjuvant chemotherapy of gastric cancer but also for the chemotherapy of various cancers such as unresectable advanced gastric cancer, pancreatic cancer, gallbladder cancer, colorectal cancer, head and neck cancer, and breast cancer in Japan. It is desired to investigate the cause of the watering eyes and to establish the effective methods of prevention and treatment for the adverse event.

Based on the hypothesis formulated from previous reports that the components originated from S-1 cause inflammation of lachrymal duct which results in dacryostenosis, the countermeasures such as providing eyedrops to dilute tears and inserting stent into the lachrymal duct of the patient with dacryostenosis are currently practiced case by case in clinical situations.

However, the previous reports are those based on the small numbers of target patients, the retrospective studies, and the patients who already suffered from the watering eyes and the disorder of lachrymal duct [7–14]. And, there are no reports which evaluated whether and which components and active modulator of S-1 were detected in the tears of the patients taking S-1, and whether their PK in tears correlate with those in plasma, except for a prospective study about the correlation between the concentrations in plasma and those in tears reported by Kim et al. [15]. But, in the report, only FT was detected in tears, while 5-FU and CDHP were not, and the influence of other S-1 components to watering eyes was not identified.

Our clinical study this time is the first one which was designed to confirm of the correlation between the concentrations of S-1 components and active modulator in plasma and those in tears in terms of FT, 5-FU, and CDHP by targeting Japanese patients who started to orally take S-1. In the study, all the FT, 5-FU and CDHP were detected both in plasma and in tears. And, it was found that there was positive correlation between the concentrations of all the FT, 5-FU, and CDHP in plasma and those in tears. It might be assumed that all the FT, 5-FU, and CDHP were the causes of inflammation which resulted in the watering eyes and the disorder of lachrymal duct, which needs further investigation on the correlation of watering eyes and the concentrations of each FT, 5-FU, and CDHP.

The finding enables us to estimate the PK in tears by measuring those in plasma, which is expected to bring great benefit to the patients, because the measurement of the PK in tears is difficult and puts substantial strain to patients. The result of this study suggested that eye drops used in a clinical practice to washout tears was probably effective for the prevention and treatment of watering eyes. Preclinical study with dog also demonstrated that artificial tears instillation can alleviate corneal surface damage induced by S-1 [16]. These findings are considered as a milestone which may facilitate to elucidate the cause of watering eyes, to establish the prevention method, and to predict the patients who are susceptible to watering eyes. We are considering it necessary to investigate the correlation between the concentrations in plasma and the occurrence of watering eyes and to clarify the cause of watering eyes in more details.

## Conclusion

There was a positive correlation between the concentrations of all the FT, 5-FU and CDHP in the tears and those in the plasma. The result is expected to facilitate the further investigation into the causes of watering eyes and the establishment of the effective methods for the prevention and the treatment.

## References

[1] Esmaeli B, Golio D, Lubecki L (2005). Canalicular and nasolacrimal duct blockage: an ocular side effect associated with the antineoplastic drug S-1. Am J Ophtalmol.

[2] Koizumi W, Narahara H, Hara T (2007). S-1 plus cisplatin versus S-1 alone for first-line treatment of advanced gastric cancer (SPIRITS trial): a phase III trial. Lancet Oncol.

[3] Yamada Y, Higuchi K, Nishikawa K (2015). Phase III study comparing oxaliplatin plus S-1 with cisplatin plus S-1 in chemotherapy-naïve patients with advanced gastric cancer. Ann Oncol.

[4] Boku N, Yamamoto S, Fukuda H (2009). Fluorouracil versus combination of irinotecan plus cisplatin versus S-1 in metastatic gastric cancer: a randomized phase 3 study. Lancet Oncol.

[5] Sakuramoto S, Sasako M, Yamaguchi T (2007). Adjuvant chemotherapy for gastric cancer with S-1, an oral fluoropyrimidine. N Engl J Med.

[6] Tabuse H, Kashiwagi H, Hamauchi S (2016). Excessive watering eyes in gastric cancer patients receiving S-1 chemotherapy. Gastric Cancer.

[7] Eiseman AS, Flanagan JC, Brooks AB (2003). Ocular surface, ocular adnexal, and lacrimal complications associated with the use of systemic 5-fluorouracil. Ophthal Plast Reconstr Surg.

[8] Kim N, Park C, Park DJ (2012). Lacrimal drainage obstruction in gastric cancer patients receiving S-1 chemotherapy. Ann Oncol.

[9] Park J-S, Ha SW, Lew H (2010). Two cases of lacrimal drainage obstruction associated with S-1 anticancer treatment. J Korean Ophthalmol Soc.

[10] Kitamura H, Miyanaga T, Shin H (2011). Investigation of epiphora following S-1 therapy. Jpn J Cancer Chemother.

[11] Shioda K, Tanabe K, Kimura S (2009). Treatment of severe canalicular blockage secondary to peroral TS-1. J Clin Ophthalmol.

[12] Kashiwagi H (2010). Ocular disorders of anticancer drugs—ocular side effects. Jpn J Cancer Chemother.

[13] Sakai J, Inoue Y, Kashiwagi H (2012). Multi-institutional survey on the lacrimal side effects of TS-1. J Clin Ophthalmol.

[14] Sasaki T, Miyashita H, Miyanaga T (2012). Dacryoendoscopic observation and incidence of canalicular obstruction/stenosis associated with S-1, an oral anticancerdrug. Jpn J Ophthalmol.

[15] Kim N, Kim JW, Baek JH et al (2017) S-1–induced lacrimal drainage obstruction and its association with ingredients/metabolites of S-1 in tears and plasma: a prospective multi-institutional study. Cancer Res Treat 10.4143/crt.2016.569 PMC5784617 28253565

[16] Kanie S, Fujieda M, Hitotsumachi T (2017). Alleviating effects of artificial tear instillation on S-1-induced ocular toxicity in dogs. J Toxicol Sci.

